# Nurses for Board Schools

**Published:** 1898-07-23

**Authors:** 


The Hospital

A Journal of

The Medical Sciences and Hospital Administration.

Vol. XXIV.—No. 617.] Edited by Sir Henry Burdett, K.C.B., and Solomon C. Smith, M.D., M.R.C.P. [July 23, 1898.

### Nurses for Board Schools.

The sanitary state and surroundings of Board Schools, especially in the poorer districts of the metropolis, are beginning to attract a considerable amount of attention in more than one quarter, and a movement has been initiated which might certainly lead to some abuses under the influence of indiscretion, and will need to be wisely guided if it is to be productive of beneficial results. An association has been formed for the purpose of providing visiting nurses for the schools, and its proceedings are under the direction of a committee of which Mr. Lyulph Stanley is chairman, which is composed partly of members of the School Board and partly of other persons, which holds its meetings in the School Board offices, and which, therefore, will be likely, in the eyes of teachers and managers, to possess at least a semi-official character and authority. In an unofficial way, as has already been noted in our columns, district nurses have already visited certain schools, but this association will now appoint as many visiting nurses as it can find funds to support; and the first of them will enter upon her duties when the schools reassemble after the summer vacation. To each nurse a certain number of schools will be allotted, and her instructions will be to visit them at regular intervals. Such small ailments as broken chilblains in winter, or wounds or sores upon exposed feet at all times, will be brought to her for dressings, together with the hundred and one trivial injuries to be found upon the bodies of dirty or neglected children. She will be expected to notice the presence of vermin in the hair or eyelashes, and the early stages of the various forms of juvenile ophthalmia, as well as any indications of the forms of eye distress which may arise from errors of refraction. She will give personal attention to the dressing of sores, and will be expected to communicate with the parents, either directly or through the teacher or the committee, in all cases in which it seems desirable that the condition of a child should be brought under medical notice and supervision. While we admit the benefits which many individual children may derive from such a nurse's help, we cannot but see that the system bristles with many difficulties. The old definition of an archdeacon was that he was a person who performed archidiaconal functions; and if the nurses will justify a definition of themselves based upon this principle, and will confine themselves to the discharge of nursing functions, there can hardly be a doubt that their services will be valuable. The chief trouble which may arise in connection with the matter is one against which the Committee ought to be forewarned, and it is the risk lest the nurses, "rendered rash by ignorance," should take upon themselves to attempt the discharge of purely medical duties. Their real business, setting aside the care of a broken chilblain or of a cut finger, will be to form a band of sentries against the insidious approaches of disease, and a band of instructors in the first principles of cleanliness and hygiene. As in warfare, the pickets fall back upon the main body as soon as an enemy is seen to be in force, so the visiting nurses must be prepared to fall back upon medical practitioners as soon as they are confronted by real illness; and here we are met at once with the defect that no such medical supports are provided by the scheme. Even district nurses, acting as they ostensibly do under the direction and supervision of medical men, and tending exclusively those who are ill and usually are under medical care, often enough find it difficult to resist the temptation to assume medical functions, and it is easy enough to see how much more strongly nurses will be drawn into playing the doctor when they see little children, who are under no medical care, suffering from little ailments which they think they can treat quite well, and when they recognise how doubtful it is that any recommendation on their part will lead the parents to provide the medical care which is required.

The temptation to do a little quacking, and the apparent reasonableness of so doing, are very likely to lead to a considerable amount of irregular medical practice among such nurses, and although not much harm may be done so long as women of common sense are found for the work, and so long as it is done by a private and entirely unofficial association, it is quite clear that it would be most improper, and indeed illegal, for such unqualified medical attendance, however trivial the ailments dealt with, to be sanctioned by a public body or paid for by public funds. If, however, the nurses will be content to do, only to do better, what the mothers ought to have done for their children, all will be well. The one great requirement, the *conditio sine quâ non* of usefulness and of success, will be the determination of the Committee, and the willingness of the nurses, to confine themselves strictly to duties which fall within the proper compass of their powers, and steadfastly to resist all temptation to engage in work, or to assume responsibilities, which require the special training of the medical profession.

